# Measurement of alveolar derecruitment in patients with acute lung injury: computerized tomography versus pressure–volume curve

**DOI:** 10.1186/cc4956

**Published:** 2006-06-22

**Authors:** Qin Lu, Jean-Michel Constantin, Ania Nieszkowska, Marilia Elman, Silvia Vieira, Jean-Jacques Rouby

**Affiliations:** 1 Surgical Intensive Care Unit Pierre Viars, Department of Anesthesiology, Assistance Publique – Hôpitaux de Paris, La Pitié-Salpêtrière Hospital, 47-83 boulevard de l'Hôpital 75013 Paris, France; 2 Surgical Intensive Care Unit, Hôtel-Dieu Hospital, Centre Hospitalo-Universitaire de Clermont Ferrand, boulevard Léon Malfreyt 63058 Clemont Ferrand cedex, France; 3 Medical Intensive Care Unit, Assistance Publique-Hôpitaux de Paris, La Pitié-Salpêtrière Hospital, 47-83 boulevard de l'Hôpital 75013 Paris, France; 4 Department of Anesthesiology of Santa Casa de Misericordia de São Paulo, Faculdade de Ciências Médicas da Santa Casa de São Paulo, Rua Dr Sesario Mota Jr, 61, Santa Cecilia/São Paulo – 01221-020 – Brazil; 5 Department of Internal Medicine, Faculty of Medicine Federal University from Rio Grande do Sul, Intensive Care Unit, Hospital de Clinicas de Porto Alegre, Rua Ramiro Barcelos, 2350 – 90035-903 Porto Alegre/Rio Grande do Sul – Brazil; 6 From the Surgical Intensive Care Unit Pierre Viars, Department of Anesthesiology, Assistance Publique-Hôpitaux de Paris, La Pitié-Salpêtrière Hospital, University School of Medicine Pierre et Marie Curie

## Abstract

**Introduction:**

Positive end-expiratory pressure (PEEP)-induced lung derecruitment can be assessed by a pressure–volume (P–V) curve method or by lung computed tomography (CT). However, only the first method can be used at the bedside. The aim of the study was to compare both methods for assessing alveolar derecruitment after the removal of PEEP in patients with acute lung injury or acute respiratory distress syndrome.

**Methods:**

P–V curves (constant-flow method) and spiral CT scans of the whole lung were performed at PEEPs of 15 and 0 cmH_2_O in 19 patients with acute lung injury or acute respiratory distress syndrome. Alveolar derecruitment was defined as the difference in lung volume measured at an airway pressure of 15 cmH_2_O on P–V curves performed at PEEPs of 15 and 0 cmH_2_O, and as the difference in the CT volume of gas present in poorly aerated and nonaerated lung regions at PEEPs of 15 and 0 cmH_2_O.

**Results:**

Alveolar derecruitments measured by the CT and P–V curve methods were 373 ± 250 and 345 ± 208 ml (*p *= 0.14), respectively. Measurements by both methods were tightly correlated (*R *= 0.82, *p *< 0.0001). The derecruited volume measured by the P–V curve method had a bias of -14 ml and limits of agreement of between -158 and +130 ml in comparison with the average derecruited volume of the CT and P–V curve methods.

**Conclusion:**

Alveolar derecruitment measured by the CT and P–V curve methods are tightly correlated. However, the large limits of agreement indicate that the P–V curve and the CT method are not interchangeable.

## Introduction

Reducing tidal volume during mechanical ventilation decreases mortality in patients with acute respiratory distress syndrome (ARDS) [1]. However, selecting the right level of positive end-expiratory pressure (PEEP) remains a difficult issue [2,3]. A recent multicenter randomized trial failed to demonstrate a decrease in mortality when a high PEEP was applied to patients with ARDS [3]. Several studies using computed tomography (CT) have suggested that the right level of PEEP should be selected according to the specific lung morphology of each individual patient, taking into consideration not only the potential for recruitment but also the risk of lung overinflation [2,4-7].

In the early 1990s, Ranieri and colleagues suggested that PEEP-induced alveolar recruitment could be measured from pressure–volume (P–V) curves [8]. Based on the physiological concept that any increase in lung volume at a given static airway pressure is due to the recruitment of previously nonaerated lung regions, PEEP-induced alveolar recruitment was defined as the increase in lung volume at a given airway pressure measured on P–V curves performed in PEEP and zero end-expiratory pressure (ZEEP) conditions [9,10]. The recruited volume measured by the P–V curve method was then found to be correlated with the increase in arterial oxygenation [9,11]. In the late 1990s, the validation of the constant flow method for measuring P–V curves [12] gave the possibility of measuring alveolar recruitment more easily at the bedside [13-15]. Consequently, the P–V curve method became a technique widely accepted by clinical researchers for assessing alveolar derecruitment [15-17]. However, this method has never been compared with another independent method. Another critical question is whether the P–V curve method can differentiate recruitment from (over)inflation.

Recently, Malbouisson and colleagues proposed a CT method for assessing PEEP-induced alveolar recruitment [18]. Alveolar recruitment was defined as the volume of gas penetrating into poorly aerated and nonaerated lung areas after PEEP. With this method, a good correlation was found between PEEP-induced alveolar recruitment and improvement of arterial oxygenation. The CT method, although considered by many as a gold standard, cannot be performed routinely and repeated easily because it requires the patient to be transported outside the intensive care unit.

We undertook a comparative assessment of the P–V curve and CT methods for measuring alveolar derecruitment after PEEP withdrawal in patients with acute lung injury (ALI) or ARDS. The aim of the study was to assess whether the P–V curve method could replace the CT method and be considered a valuable clinical tool at the bedside.

## Materials and methods

### Study design

After approval had been obtained from the Ethical Committee, and informed consent from the patients' next-of-kin, 19 patients with ALI/ARDS [19] were studied prospectively. Patients with untreated pneumothorax and bronchopleural fistula were excluded. Patients were ventilated in a volume-controlled mode with tidal volumes of 7.7 ± 1.8 ml/kg with a Horus ventilator (Taema, Antony, France). All patients were monitored with a fiber-optic thermodilution pulmonary artery catheter (CCO/SvO_2_/VIP TD catheter Baxter Healthcare co, Irvine, CA, USA) and radial or femoral arterial catheters.

After one hour of mechanical ventilation at a PEEP of 15 cmH_2_O, each patient was transported to the Department of Radiology. All patients were anesthetized and paralyzed during the study. Cardiorespiratory parameters at a PEEP of 15 cmH_2_O were recorded on a Biopac system (Biopac System Inc. Goleta, CA, USA) [20] and a P–V curve of the respiratory system at a PEEP of 15 cmH_2_O was measured with the low constant flow method (9 L/minute) [12]. Scanning of the whole lung at a PEEP of 15 cmH_2_O was performed as described previously [18]. Contiguous axial CT sections 10 mm thick were acquired after clamping the connecting piece between the Y piece and the endotracheal tube. During acquisition, airway pressure was monitored to ensure that a PEEP of 15 cmH_2_O was actually applied. The patient was then disconnected from the ventilator, and the change of end-expiratory lung volume (ΔEELV) resulting from PEEP withdrawal was measured with a calibrated pneumotachograph. P–V curve, CT scan and cardiorespiratory measurements in ZEEP conditions were performed immediately after disconnecting maneuvers. Between each measurement, mechanical ventilation at a PEEP of 15 cmH_2_O was resumed to standardize lung volume history. In seven patients, the same measurements in ZEEP were performed at the end of a 15-minute period of mechanical ventilation without PEEP.

**Figure 1:**
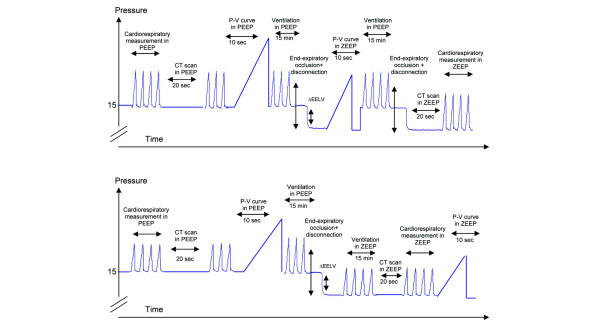
Illustration of the time course of the protocol. The upper panel represents the time course of the protocol for 12 patients for whom a computed tomography (CT) scan and pression–volume (P–V) curve in zero end-expiratory pressure (ZEEP) were acquired immediately after positive end-expiratory pressure (PEEP) withdrawal. The lower panel represents the time course of the protocol for 7 patients for whom a CT scan and P–V curve in ZEEP were acquired after 15 minutes of mechanical ventilation without PEEP. End-expiratory occlusion is defined as occlusion of the connecting piece between the Y piece and the endotracheal tube at end expiration; disconnection is defined as PEEP withdrawal, the patient being disconnected from the ventilator. ΔEELV, decrease in end-expiratory lung volume resulting from PEEP withdrawal measured by pneumotachography after the disconnecting maneuver.

The time course of the protocol is summarized in Figure 1.

### Cardiorespiratory measurements

In each patient, cardiac output, systemic arterial pressure, right atrial pressure, pulmonary artery pressure, pulmonary capillary wedge pressure and airway pressure were recorded continuously with the Biopac system. Fluid-filled transducers were positioned at the midaxillary line and connected to the different lines of the pulmonary artery catheter. Cardiac filling pressures were measured at end expiration and averaged over five cardiac cycles. Pulmonary shunt and systemic and pulmonary vascular resistances were calculated from standard formula. Expired CO_2 _was continuously recorded and measured with an infrared capnometer, and the ratio of alveolar dead space to tidal volume (V_DA_/V_T_) was calculated from the equation V_DA_/V_T _= 1 - PetCO_2_/PaCO_2_, where PetCO_2 _is end-tidal CO_2 _measured at the plateau of the expired CO_2 _curve and PaCO_2 _is arterial partial pressure of CO_2_. The compliance of the respiratory system was calculated by dividing the tidal volume by the plateau pressure minus the intrinsic PEEP.

### CT measurements of alveolar derecruitment and changes in functional residual capacity resulting from PEEP withdrawal

**Figure 2:**
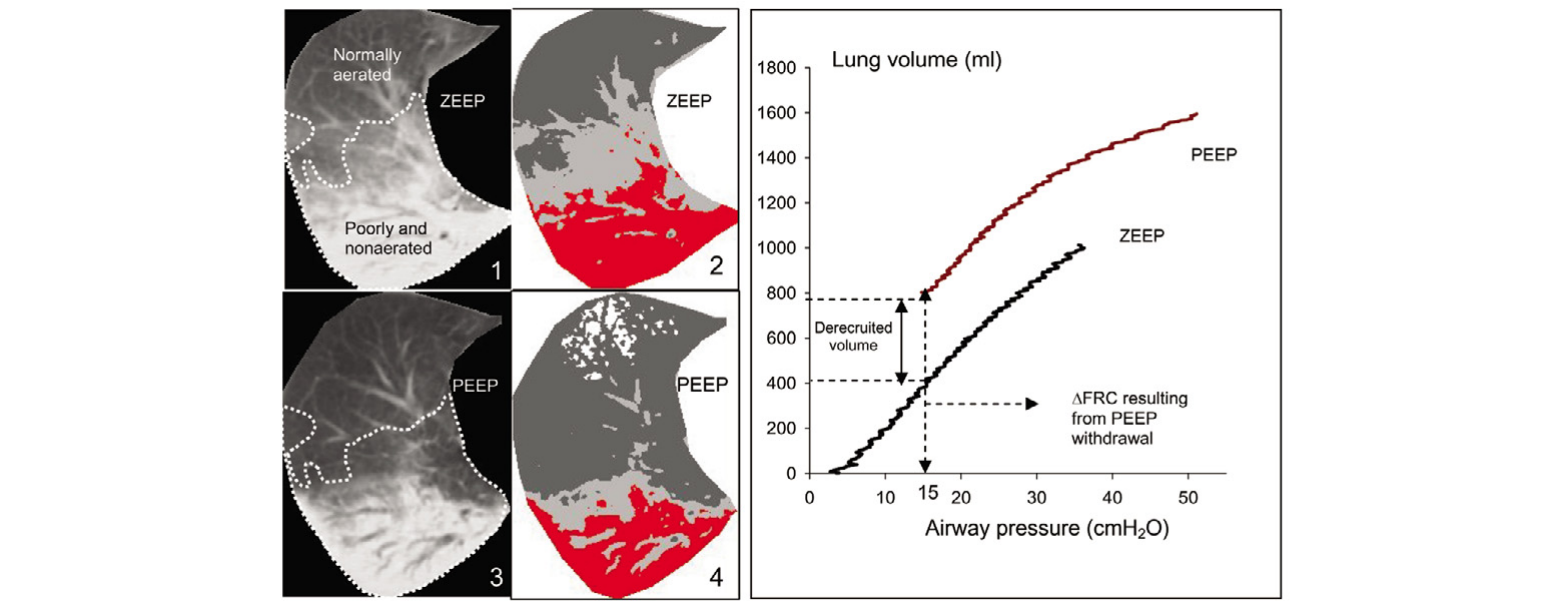
Assessment of alveolar derecruitment by computed tomography (left panel) and pressure-volume curves (right panel). Image 1 shows a computed tomography (CT) section representative of the whole lung obtained at zero end-expiratory pressure (ZEEP). The dashed line separates poorly aerated and nonaerated lung areas (which appear in light gray and red, respectively, on image 2) from normally aerated lung areas (colored in dark gray on image 2 by a color-encoding system included in Lungview). Image 3 shows the same CT section obtained at a positive end-expiratory pressure (PEEP) of 15 cmH_2_O. The delineation performed at ZEEP has been transposed on the new CT section in accordance with anatomical landmarks such as divisions of pulmonary vessels. Image 4 shows the same CT section with the color-encoding system, the overinflated lung areas appearing in white. Alveolar derecruitment was defined as the decrease in gas volume in poorly aerated and nonaerated lung regions after PEEP withdrawal. In the right panel, the pressure-volume (P–V) curves of the total respiratory system measured at ZEEP and a PEEP of 15 cmH_2_O are represented. After determining the decrease in total gas volume resulting from PEEP withdrawal (ΔFRC), ΔFRC was added to each volume for constructing the P–V curve in PEEP conditions. The two curves were then placed on the same pressure and volume axis. Derecruitment volume was identified by a downward shift of the ZEEP P–V curve compared with the PEEP P–V curve and computed as the difference in lung volume between PEEP and ZEEP at an airway pressure of 15 cmH_2_O.

CT analysis was performed on the entire lung from the apex to the diaphragm as described previously [18]. In a first step, the two CT sections obtained in ZEEP and PEEP conditions corresponding to the same anatomical level were matched and displayed simultaneously on the screen of the computer (Figure 2). Each CT section obtained in ZEEP conditions was shown on the screen of the computer with the use of a color-encoding system integrated in the Lungview^® ^software. Nonaerated voxels (CT attenuation between -100 and +100 Hounsfield units (HU)) were colored in red, poorly aerated voxels (CT attenuation between -500 and -100 HU) in light gray, and normally aerated voxels (CT attenuation between -500 and -900 HU) in dark gray. Overinflated voxels (CT attenuations between -900 and -1,000 HU) were colored in white. As shown in Figure 2, the color encoding served to separate two regions of interest on each CT section: normally aerated lung regions, and poorly or nonaerated lung regions. In a second step, by referring to anatomical landmarks, the limit between the two regions of interest delineated on the CT section in ZEEP conditions was manually redrawn on the CT section in PEEP conditions. During the regional analysis, two CT sections obtained in PEEP often corresponded to a single CT section obtained in ZEEP conditions, as attested by the anatomical landmarks (divisions of bronchial and pulmonary vessels). In such a situation, the region of interest manually delineated on the ZEEP CT section was manually delineated on the two corresponding CT sections obtained in PEEP conditions. In each of the two regions of interest delineated in ZEEP and PEEP conditions – namely, normally aerated lung region, and poorly aerated and nonaerated lung regions – the volumes of gas and tissue were computed from the following equations [18], in which CT number is the CT attenuation of the compartment analyzed:

volume of the voxel = (size of the pixel)^2 ^× section thickness     (1)

total lung volume = number of voxels × volume of the voxel     (2)

volume of gas = (-CT number/1,000) × total volume, if the compartment considered has a CT number below 0 (volume of gas = 0 if the compartment considered has a CT number above 0)     (3)

volume of lung tissue = (1 + CT number/1,000) × total volume, if the compartment considered has a CT number below zero     (4)

volume of lung tissue = number of voxels × volume of the voxel, if the compartment considered has a CT number above zero     (5)

The change in functional residual capacity resulting from PEEP withdrawal (ΔFRC) was computed as the difference in total volume of gas in the whole lung between PEEP and ZEEP. Alveolar derecruitment was defined as the difference in gas volume in poorly aerated and nonaerated lung regions between PEEP and ZEEP. The changes in gas volume resulting from PEEP withdrawal in normally aerated lung regions characterized by CT attenuations between -500 and -900 HU were computed separately (Figure 2). As described previously [21], the distribution of the loss of lung aeration in each patient (lung morphology) was classified as diffuse, patchy, and lobar on the basis of the distribution of CT attenuations at ZEEP.

### Pneumotachographic measurement of changes in end-expiratory lung volume resulting from PEEP withdrawal

ΔEELV was measured with a heated pneumotachograph (Hans Rudolph Inc, Kansas City, KA, USA) positioned between the Y piece and the connecting piece. The pneumotachograph was previously calibrated with a supersyringe filled with 1,000 ml of air. The precision of the calibration was 3%. The respiratory tubing connecting the endotracheal tube to the Y piece of the ventilatory circuit was occluded by a clamp at an end-expiratory pressure of 15 cmH_2_O while the ventilator was disconnected from the patient. This occlusion was performed after a prolonged expiration obtained by decreasing the respiratory rate to 5 breaths/minute. The clamp was then released and the exhaled volume measured by the pneumotachograph was recorded on the Biopac system. The total duration from PEEP withdrawal to reconnection of the ventilator to the patient was 7.4 ± 0.4 s.

### Measurement of alveolar derecruitment by P–V curves

P–V curves of the respiratory system were acquired with the specific software of the Horus ventilator – low constant flow technique [12] – and recorded with the Biopac system. During insufflation, the maximum peak airway pressure was limited to 50 cmH_2_O. Data pairs of airway pressure and volume of the P–V curves in ZEEP and PEEP conditions recorded on the computer were fitted to a sigmoid model as proposed by Venegas and colleagues [22]. The lower and upper inflection points as well as the slope of the linear part of the curve between lower and upper inflection points were computed from inspiratory P–V curves in ZEEP conditions.

Because the Horus ventilator was not equipped with a specific software measuring alveolar derecruitment directly, alveolar derecruitment resulting from PEEP withdrawal was measured from the data recorded on the computer with the help of Microsoft Excel files as follows: first, ΔFRC was added to each volume of the P–V curve in PEEP conditions; then the P–V curves in ZEEP and PEEP conditions were placed on the same volume axis. Derecruited volume was computed as the difference in lung volume between PEEP and ZEEP at an airway pressure of 15 cmH_2_O [10] (Figure 2).

### Statistical analysis

Data are expressed as means ± SD or as median (range) depending on the data distribution. Cardiorespiratory and CT variables were compared before and after the administration of PEEP with the use of a paired Student *t *test or a Wilcoxon test. All correlations were made by linear regression. Agreement between CT and P–V curve methods was tested with the Bland and Altman method [23]: the bias was expressed as the mean difference of derecruited volume between the P–V curve method and the average value of the P–V curves and CT methods; the limits of agreement were defined as 2 SD. The statistical analysis was performed with Sigmastat 3.1 (Systat Software Inc., Point Richmond, CA, USA). The statistical significance level was fixed at *p *= 0.05.

## Results

### Patients

Nineteen consecutive patients with ALI/ARDS (2 females and 17 males; age 48 ± 17 yrs) were studied. ALI/ARDS was related to postoperative pulmonary infection (*n *= 10), bronchopulmonary aspiration in the postoperative period (*n *= 5), lung contusion (*n *= 3), and extracorporeal circulation (*n *= 1). Three patients had diffuse, nine patchy and seven lobar loss of lung aeration. The delay between the onset of ALI/ARDS and inclusion in the study was 3 days (range, 1 to 10 days). The lung injury severity score [24] was 2.3 ± 0.7. Ten patients had septic shock requiring norepinephrine (noradrenaline). The overall mortality rate was 32%.

### Cardiorespiratory changes and P–V curves in ZEEP and PEEP conditions

As shown in Table 1, PEEP withdrawal resulted in a significant decrease in arterial partial pressure of oxygen (PaO_2_) and pulmonary capillary wedge pressure, and a significant increase in pulmonary shunt, PaCO_2_, slope of the P–V curve, mean arterial pressure, and cardiac index.

Sixteen patients had a lower inflection point and 17 an upper inflection point on their P–V curves in ZEEP: these were at 9.2 ± 4.8 cmH_2_O (range 3 to 16 cmH_2_O) and 28.1 ± 5.4 cmH_2_O (19 to 40 cmH_2_O), respectively.

### Comparison of PEEP-induced changes in end-expiratory lung volume measured by pneumotachography and functional residual capacity measured by CT

In the 12 patients in whom CT sections at ZEEP were acquired immediately after the disconnecting maneuver, ΔFRC and ΔEELV were similar (1,054 ± 352 ml versus 1,022 ± 315 ml). In the 7 patients in whom CT sections at ZEEP were acquired 15 minutes after the disconnecting maneuver, ΔFRC was significantly greater than ΔEELV (1,167 ± 230 versus 1,028 ± 200 ml, *p *= 0.03). Very probably, the period of 15 minutes of mechanical ventilation without PEEP induced an additional time-dependent derecruitment.

### Comparison of alveolar derecruitment measured by the CT and P–V curve methods

CT analysis showed that PEEP withdrawal induced a significant increase in poorly aerated and nonaerated lung volumes and a decrease in normally aerated lung volume (Table 2). One-third of the decrease in FRC resulting from PEEP withdrawal was related to lung derecruitment, the other two-thirds being caused by the deflation of normally aerated lung regions (Table 3). In PEEP conditions, lung overinflation of between 7 and 446 ml was observed in 10 patients.

**Figure 3:**
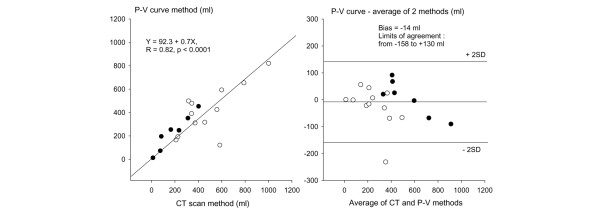
Comparison of alveolar derecruitment assessed by the computed tomography and pressure–volume curve methods. In the left panel, the linear correlation existing between the two methods is represented. In the right panel, the agreement between the two methods is represented with the Bland and Altman analysis. Open circles indicate 12 patients in whom alveolar derecruitment was measured by both methods immediately after the disconnecting maneuver; closed circles identify seven patients in whom alveolar derecruitment was measured by both methods 15 minutes after PEEP withdrawal. The bias was expressed as the mean difference between the derecruited volume measured by the P–V curve method and the average value of the two methods. The limits of agreement were defined as 2 SD.

As shown in Figure 3, alveolar derecruitment measured by the P–V curve method was tightly correlated with alveolar derecruitment measured by the CT scan method. The derecruited volume measured by the P–V curve method had a bias of -14 ml and limits of agreement between -158 and +130 ml in comparison with the average derecruited volume of the CT and P–V curve methods. The decrease in gas volume in the normally aerated lung regions resulting from PEEP withdrawal measured by CT was tightly correlated with lung volume measured at an airway pressure of 15 cmH_2_O on the P–V curve performed in ZEEP conditions (*y *= 51.6 + 0.95*x*, *R *= 0.90, *p *< 0.0001).

**Figure 4:**
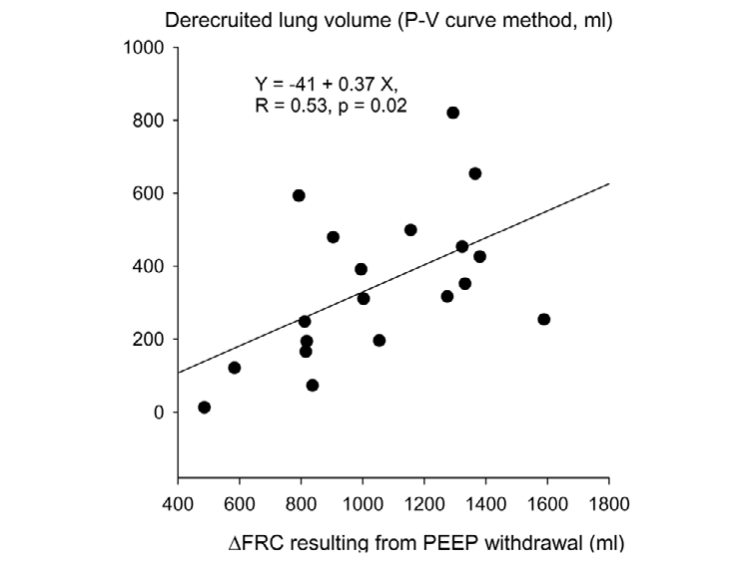
Relationship between ΔFRC and alveolar derecruitment measured by the P–V curve method. ΔFRC, change in functional residual capacity; PEEP, positive end-expiratory pressure

ΔFRC resulting from PEEP withdrawal was weakly correlated with alveolar derecruitment measured by the P–V curve method (Figure 4). The change in nonaerated lung volume resulting from PEEP withdrawal measured by CT was not correlated to the derecruited volume measured by the P–V curve method (*R *= 0.4, *p *= 0.07).

## Discussion

This study shows a statistically tight correlation between alveolar derecruitment measured by the P–V curve and CT methods. However, the large limits of agreement indicate that the P–V curve method cannot replace the CT method.

### Comparison of changes in functional residual capacity measured by CT and changes in end-expiratory lung volume measured by pneumotachography

Alveolar derecruitment resulting from PEEP withdrawal rather than recruitment induced by PEEP implementation was measured in the present study first and foremost for safety and methodological reasons. Each patient enrolled in the study was ventilated with PEEP at inclusion and the clinician in charge considered that PEEP had to be maintained during the transportation to the Department of Radiology. Ventilation with PEEP was therefore considered as the control condition. In addition, ΔEELV, an indispensable parameter for constructing P–V curves in PEEP conditions, can be measured by pneumotachography only during a PEEP releasing maneuver, which corresponds to a derecruitment maneuver.

After PEEP withdrawal, lung derecruitment continues. This study was initially designed for measuring immediate and time-dependent derecruitment after PEEP withdrawal as recommended previously [8,9]. This is the reason that seven patients underwent CT scan and P–V curve at ZEEP, 15 minutes after PEEP withdrawal. ΔEELV, measured by pneumotachography immediately after PEEP withdrawal, was initially used for constructing the P–V curve at PEEP. However, after completing the CT analysis of the seven patients, we found that ΔFRC computed from CT scan data was 15% greater than ΔEELV measured by pneumotachography. In other words, a 15-minute period of mechanical ventilation at ZEEP had induced an additional lung derecruitment that could not be measured by pneumotachography. If, as initially planned, we had used ΔEELV measured by pneumotachography for constructing the P–V curve at PEEP, alveolar derecruitment measured by the P–V curve method would have been underestimated. This is why, in the present study, ΔFRC rather than ΔEELV was used in the construction of the P–V curve at PEEP.

If ΔEELV is measured by direct spirometry (pneumotachography, hot wire, or any other technique), the existence of a time-dependent lung derecruitment imposes the requirement to perform the measurement immediately after a PEEP releasing maneuver. Our main objective was to validate the P–V curve method, the only method that might have a bedside application. To standardize the conditions for measuring ΔFRC and ΔEELV, P–V curves and CT scans at ZEEP were measured immediately after PEEP withdrawal in an additional group of 12 patients. Measurement of time-dependent lung derecruitment requires the measurement of changes in FRC (CT and gas dilution techniques). As far as assessment of time-dependent lung derecruitment is concerned, the possibility of measuring FRC provided on some recent ventilators is of potential interest, especially if coupled with the possibility of measuring P–V curves [25].

### Comparison of alveolar derecruitment measured by the CT and P–V curve methods

When PEEP is applied to lungs whose loss of aeration is heterogeneously distributed, a part of the gas entering the respiratory system penetrates into poorly aerated and nonaerated lung regions, whereas another part (over)inflates previously aerated ones. Only the gas penetrating into poorly aerated and nonaerated lung regions can be considered as lung recruitment. Quite often it represents a small part of PEEP-induced increase in lung volume and (over)inflation largely predominates over recruitment [26]. The same reasoning can be applied to alveolar derecruitment resulting from PEEP withdrawal. In the present study, lung derecruitment represented only one-third of total changes in gas volume. The other two-thirds was due to gas volume loss in normally aerated lung regions. This is the reason why the computed tomographic method developed by Malbouisson and colleagues for measuring PEEP-induced alveolar recruitment is based on a separate analysis of the gas penetrating into poorly aerated and nonaerated lung regions and into normally aerated lung areas [18].

Proposed in the late 1990s [13,14,27], the P–V curve method is based on the physiological concept that, at a given static airway pressure, any increase in gas volume after PEEP administration is due to the reaeration of previously collapsed lung units [9]. However, this hypothesis may be invalidated by the heterogeneity and complexity of the reaeration process after an increase in airway pressure. In most ARDS lungs, nonaerated and normally aerated lung areas coexist at ZEEP. Previous CT data [18,26,28] demonstrated that alveolar recruitment of nonaerated lung regions may be associated with inflation and overinflation of previously normally aerated lung areas. A recent CT study, performed during a P–V curve maneuver, demonstrated that, during the inflation of the lungs, alveolar recruitment occurs simultaneously with inflation and overinflation of previously aerated lung regions [29]. One essential question is whether the P–V curve method can differentiate between recruitment and (over)inflation.

**Figure 5:**
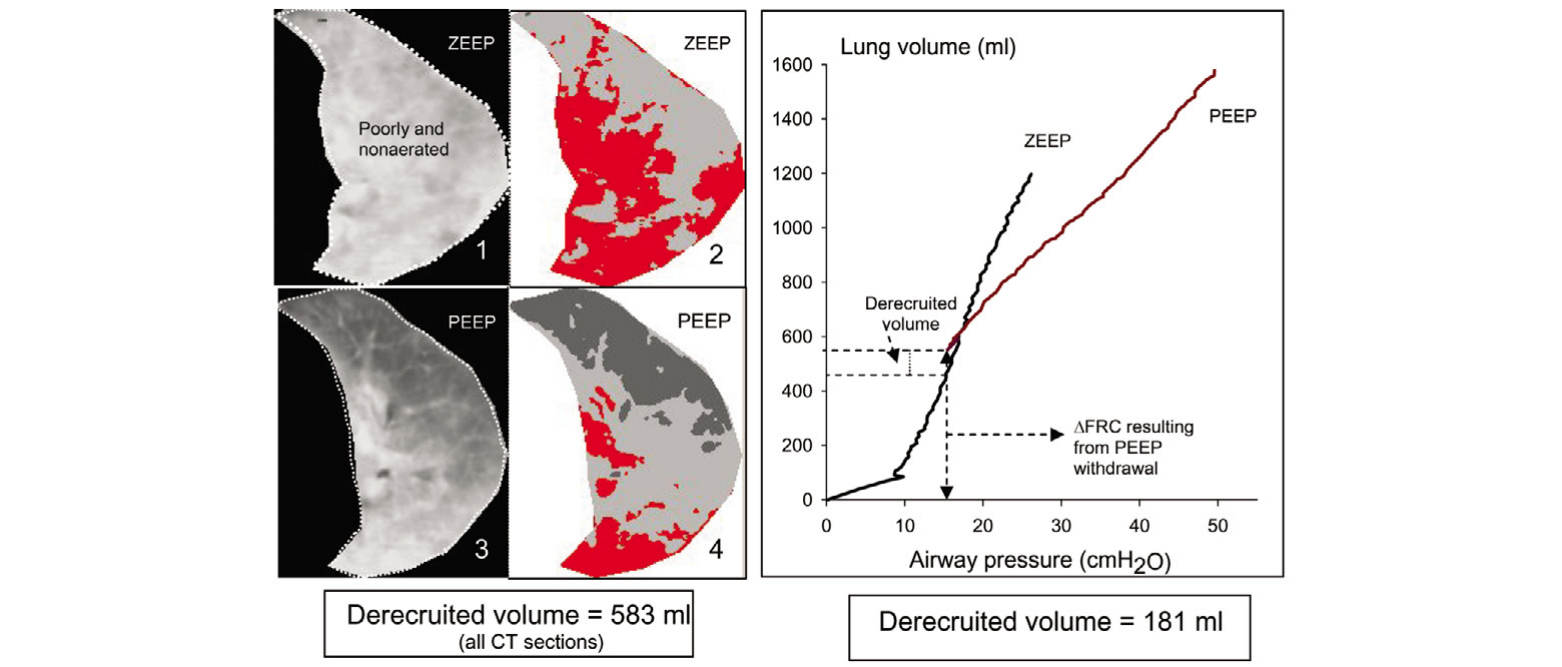
CT sections and P–V curves in a patient with diffuse loss of lung aeration. Image 1 shows a computed tomographic (CT) section representative of the whole lung obtained at zeron end-exoiratory pressure (ZEEP). The dashed line delineates the poorly aerated and nonaerated lung areas, which appear in light gray and red, respectively, on image 2 in accordance with a color-encoding system included in Lungview. Normally aerated lung areas are not observed and the delineation corresponds to the lung parenchyma present on the CT section. Image 3 shows the same CT section obtained at a positive end-expiratory pressure (PEEP) of 15 cmH_2_O. Image 4 shows the same CT section to which the color encoding has been applied, the normally aerated areas appearing in dark gray. In this patient without any normally aerated lung areas at ZEEP, alveolar derecruitment computed by the CT scan method is equal to the total decrease in functional residual capacity (ΔFRC = 583 ml). Because both CT and the pressure-volume (P–V curve) at ZEEP were acquired immediately after PEEP withdrawal, alveolar derecruitment is also equal to changes in end-expiratory lung volume measured by pneumotachography (596 ml). The P–V curve method markedly underestimates PEEP-induced alveolar derecruitment measured by the CT method.

CT derecruitment resulting from PEEP withdrawal was significantly and tightly correlated with the derecruitment measured by the P–V curve method. There was also a weak, but statistically significant, correlation between lung derecruitment measured by P–V curve and ΔFRC resulting from PEEP withdrawal. However, the large limits of agreement between both methods suggest that the P–V curve is not interchangeable with the CT scan method. A recent electrical impedance tomography study has demonstrated that, during tidal inflation, the normally aerated lung is expanded earlier than the consolidated lung [30,31]. Our result confirms that the initial portion of the P–V curve in ZEEP is essentially influenced by the inflation of previously normally aerated lung regions. When the initial increase in lung volume measured at an airway pressure of 15 cmH_2_O on the P–V curve in ZEEP conditions consists exclusively of the inflation of normally aerated lung areas, the derecruitment resulting from PEEP withdrawal measured by the CT and P–V curves is the same. However, if the initial increase in lung volume measured at an airway pressure of 15 cmH_2_O on the P–V curve in ZEEP consists partly or exclusively of reaeration of poorly aerated or nonaerated lung areas (lung recruitment), then the derecruitment resulting from PEEP withdrawal measured by P–V curves underestimates CT derecruitment. Such a condition is illustrated by a patient in the present study in whom CT alveolar derecruitment was underestimated by 69% by the P–V curve method. At ZEEP, the patient had a bilateral and diffuse loss of aeration without any normally aerated lung areas (Figure 5). Lung derecruitment measured by CT immediately after PEEP withdrawal was equal to ΔFRC and ΔEELV because each expired milliliter contributed to an increase in poorly aerated and nonaerated lung regions [5,32]. Therefore, discarding the lung volume corresponding to an airway pressure of 15 cmH_2_O on the ZEEP P–V curve leads to an underestimate of lung derecruitment.

Previous studies have suggested that measuring lung derecruitment by the P–V curve method immediately after PEEP withdrawal might result in an underestimate of overall lung derecruitment by ignoring the additional derecruitment occurring with time [10,33]. The present study provides convincing evidence that time-dependent lung derecruitment can be correctly assessed by the P–V curve method at a single condition: an accurate measurement of changes in FRC either by CT or by the gas dilution technique. Again, the recent possibility offered by recent ventilators of measuring FRC by the gas dilution technique and P–V curves by the low flow inflation technique offers an attractive opportunity of measuring lung recruitment and derecruitment at the bedside.

In fact, the CT and P–V curve methods do not measure exactly the same lung derecruitment. The CT method measures end-expiratory lung derecruitment, whereas the P–V curve method measures the difference in volume between the P–V curve in PEEP and ZEEP conditions at a given elastic pressure. Ideally, the validation of the P–V curve method by the CT method should have implied the acquisition of CT sections not only in PEEP conditions but also during an insufflation maneuver of the P–V curve in ZEEP conditions at a pressure of 15 cmH_2_O. End-inspiratory lung volume at this pressure should have been subtracted from total changes in FRC resulting from PEEP withdrawal. Unfortunately, CT technology does not permit the acquisition of CT sections of the whole lung at a fixed inspiratory pressure during a quasi-static inflation maneuver. Another confounding factor that might interfere with alveolar derecruitment measured with the P–V curve method could be an alteration of the chest wall elastance. It is also well known that atelectasis of caudal and dependent lung regions resulting from an increase in intra-abdominal pressure induces a rightward shift of the P–V curve [34]. Whether such a condition influences the alveolar derecruitment computed from respiratory and P–V curve methods remains to be determined.

## Conclusion

The present study demonstrates that lung derecruitment derived from P–V curves is tightly correlated with lung derecruitment measured by CT. As a result, it provides useful information on PEEP-induced lung derecruitment at the bedside. However, the P–V curve method measures a lung derecruitment that is different from the CT lung derecruitment measured in true static end-expiratory conditions and can be influenced by aeration changes occurring during the initial part of the inflation P–V curve performed in ZEEP conditions.

## Key messages

• 	Computed tomography is a gold standard for the assessment of lung derecruitment in patients with acute lung injury.

• 	The pressure–volume curve can measure lung derecruitment at the bedside.

• 	Lung derecruitment resulting from posivite end-expiratory pressure measured by two methods is tightly correlated, but the derecruited volume measured by the pressure–volume curve has a large limits of agreement in comparison with the average volume of the both methods.

• 	The pressure–volume curve cannot replace the computed tomography method.

## Abbreviations

ALI = acute lung injury; ARDS = acute respiratory distress syndrome; CT = computed tomography; ΔEELV = changes in end-expiratory lung volume measured by pneumotachography; ΔFRC = change in functional residual capacity measured by the computed tomography method; HU = Hounsfield unit; PaCO_2 _= arterial partial pressure of CO_2_; PEEP = positive end-expiratory pressure; P–V = pressure–volume; ZEEP = zero end-expiratory pressure.

## Competing interests

The authors declare that they have no competing interests.

## Authors' contributions

QL performed the study and drafted the manuscript. JMC and AN participated in the study and in the study analysis. ME and SV participated in the acquisition of the data for the study. JJR participated in the design of the study and helped to draft the manuscript. All authors read and approved the final manuscript.

## Figures and Tables

**Table 1:** Cardiorespiratory parameters of 19 patients at PEEPs of 15 cmH_2_O and 0

Parameter	PEEP	ZEEP	*p*
PaO_2 _(mmHg)	213 ± 83	147 ± 80	<0.0001
Qs/Qt (%)	30 ± 6	39 ± 9	<0.0001
PaCO_2 _(mmHg)	43 ± 8	46 ± 8	0.006
VD_A_/VT (%)	29 ± 11	33 ± 12	NS
Crs (ml cmH_2_O^-1^)	56 ± 26	48 ± 14	NS
Slope (ml cmH_2_O^-1^)	53 ± 21	69 ± 26	0.003
PEEPi (cmH_2_O)	2.2 ± 1.1	0.8 ± 1.1	0.001
MAP (mmHg)	84 ± 13	92 ± 15	0.006
SVRI (dyn s^-1 ^cm^-5 ^m^2^)	1,767 ± 748	1,916 ± 1,114	NS
MPAP (mmHg)	28 ± 8	25 ± 9	NS
PVRI (dyn s^-1 ^cm^-5 ^m^2^)	345 ± 164	289 ± 174	NS
PCWP (mmHg)	14 ± 3	11 ± 4	0.02
CI (l minute^-1 ^m^-2^)	3.7 ± 1.8	4.3 ± 1.8	0.03

CI, cardiac index; Crs, respiratory compliance; MAP, mean arterial pressure; MPAP, mean pulmonary arterial pressure; NS, not significant; PaCO_2_, arterial partial pressure of CO_2_; PaO_2_, arterial partial pressure of oxygen; PCWP, pulmonary capillary wedge pressure; PEEPi, intrinsic positive end-expiratory pressure; PVRI, pulmonary vascular resistance index; Qs/Qt, pulmonary shunt; slope, respiratory inflation compliance; SVRI, systemic vascular resistance index; VD_A_/VT, alveolar deadspace; ZEEP, zero end-expiratory pressure; PEEP, positive end-expiratory pressure. Data are expressed as means ± SD.

**Table 2:** Computed tomographic analysis of degrees of lung aeration of the whole lung

Parameter	PEEP	ZEEP	*p*
Lung volume, gas + tissue (ml)	3,372 ± 686	2,283 ± 549	<0.001
Functional residual capacity (ml)	2,035 ± 594	992 ± 450	<0.001
Volume of tissue (ml)	1,344 ± 315	1,296 ± 328	0.015
Overinflated lung volume (ml)	51 ± 121 (0–508)	4 ± 11(0–45)	<0.001
Normally aerated lung volume (ml)	2,476 ± 649	1,133 ± 640	<0.001
Poorly aerated lung volume (ml)	394 ± 224	597 ± 280	0.002
Nonaerated lung volume (ml)	451 ± 275 (123–1,213)	549 ± 342 (165–1,452)	0.001

ZEEP, zero end-expiratory pressure; PEEP, positive end-expiratory pressure of 15 cmH_2_O. Results in parentheses are ranges.

**Table 3:** Separate regional computed tomographic analysis of normally aerated/poorly aerated and nonaerated lung regions

Parameter	PEEP	ZEEP	*p*
Regional analysis performed in poorly aerated and nonaerated lung regions			
Lung volume, gas + tissue (ml)	1,438 ± 582	1,068 ± 424	< 0.001
Volume of gas (ml)	561 ± 325	188 ± 109	< 0.001
Volume of tissue (ml)	877 ± 361	880 ± 376	NS
Regional analysis performed in normally aerated lung regions			
Lung volume, gas + tissue (ml)	1,940 ± 985	1,220 ± 696	< 0.001
Volume of gas (ml)	1,474 ± 779	803 ± 515	< 0.001
Volume of tissue (ml)	466 ± 212	416 ± 190	< 0.001

ZEEP, zero end-expiratory pressure; PEEP, positive end-expiratory pressure of 15 cmH_2_O.
